# Anterior Intercostal Artery Perforator Flap for Delayed Implant Exposure Salvage After Prepectoral Breast Reconstruction

**DOI:** 10.1093/asjof/ojag113

**Published:** 2026-06-20

**Authors:** Rossella Gioco, Francesco Caruso, Gaetano Castiglione, Mariagloria Marino, Ada Cinquerrui, Irene Cannata, Debora Simona Fichera, Nicola Francesca Musmeci, Konstantina Balafa, Marco Latino, Angelo Rosa, Giuseppe Catanuto, Dario Virzì

## Abstract

The anterior intercostal artery perforator (AICAP) flap has emerged as a viable option in breast reconstructive surgery for salvaging implants compromised by infection or radiotherapy. However, its application in delayed implant exposure following prepectoral reconstruction remains limited in the literature. The authors report the case of a 54-year-old woman who presented with localized infection and implant exposure following nipple-sparing mastectomy and prepectoral breast reconstruction with a silicone implant. Oncologic recurrence was excluded. Conventional conservative therapy failed, and the defect was successfully managed with an AICAP flap for implant salvage. This case highlights the feasibility of this technique as a less invasive alternative to avoid implant removal and secondary reconstruction, particularly in selected patients without extensive soft tissue compromise.

Level of Evidence: 5 (Therapeutic)

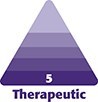

Implant-based breast reconstruction is widely performed after mastectomy, with an increasing trend toward prepectoral placement.^1^ However, complications such as infection, skin necrosis, and implant exposure (IE) may jeopardize reconstructive outcomes.^2,3^

IE is an uncommon but serious event that carries pathological, psychological, and financial consequences for patients.^4,5^ When IE occurs, the standard approach is to remove the implant and delay breast reconstruction.^4,6,7^

In selected cases of mild exposure or infection, however, implant salvage may represent a valuable alternative, because many patients are reluctant to undergo explantation. In fact, if there is no contamination of the prosthetic device, salvage techniques prove to be effective and show encouraging success rates, playing a critical role in avoiding implant loss.^8,9^ Nevertheless, clear indications and standardized strategies for implant salvage in delayed exposure after prepectoral reconstruction remain insufficiently defined in the current literature.

Upon the detection of IE, direct skin closure is not always feasible, and in such cases, the AICAP flap harvested from the inframammary fold (IMF) provides additional tissue with minimal aesthetic impact.^8,9^ This flap is well suited for covering defects in the lower breast and for supporting thin mastectomy flaps. Although the anterior intercostal artery perforator (AICAP) flap has been previously described for breast reconstruction and salvage procedures, reports focusing on its use in delayed IE following prepectoral reconstruction are limited. Therefore, this case report aims to illustrate the clinical rationale and surgical application of the AICAP flap in this specific reconstructive scenario, with a late exposure of a polyurethane (PU)-coated implant used for prepectoral reconstruction. In our experience, the “Velcro effect” and the adherence of the PU surface likely prevented further enlargement of the soft tissue defect, whereas the size of the flap was adequate to allow preservation of the primary breast volume.^10^

We present the case of a woman who developed IE 3 years after nipple-sparing mastectomy and immediate prepectoral reconstruction with a silicone implant. The defect was successfully managed with an AICAP flap and immediate implant replacement, with no complications observed at 6-month follow-up, and the patient is satisfied about the aesthetic result.

## CASE PRESENTATION

A 54-year-old woman with a history of right-sided breast cancer underwent nipple-sparing mastectomy in December 2021, followed by immediate prepectoral reconstruction using a 560 mL POLYTECH Opticon MHS PU-coated silicone implant. She was an active smoker and was on adjuvant endocrine therapy with letrozole. The patient did not undergo adjuvant radiotherapy. No acellular dermal matrix (ADM) was used during the initial prepectoral reconstruction.

Approximately 3 years postoperatively, she presented with acute inflammation of the dermoepidermal flap in the lower quadrants of the right breast. Initial management with systemic and topical anti-inflammatory therapy was insufficient. After a few weeks, an ulcerated lesion of ∼0.3 cm developed because of skin necrosis in the lower inner quadrant, leading to IE. A biopsy of the ulcerated area was performed to exclude local recurrence, and histopathological examination confirmed the absence of malignancy. There were no systemic signs of infection, and cultures were negative. The exposure was considered multifactorial, likely related to chronic smoking, progressive thinning of the mastectomy flap, and local ischemia rather than active infection or malignancies, which were preliminarily excluded. Conservative treatment with oral clindamycin (300 mg every 8 h) and advanced wound care failed, and the lesion progressed to an inflammatory area of ∼3.0 cm in diameter with a central defect measuring 15 mm, resulting in exposure of the implant. After evaluating treatment options, we proceeded with implant salvage using a fully islanded AICAP flap rotated superiorly.

### Surgical Technique

**Figure 1.**
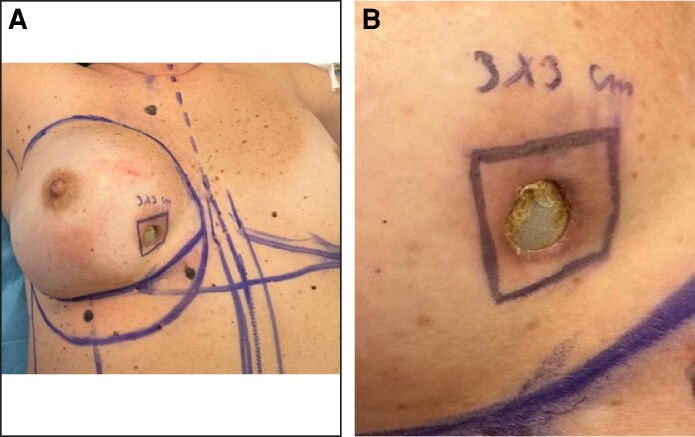
Preoperative clinical presentation in a 54-year-old female patient. (A) Preoperative planning and visualization of the perforators along the inframammary fold (the 2 lower dots, the third isolated dot indicates the side of the surgery), flap design outlining the skin island and planned arc of rotation. (B) Focus on the ulcerated lesion with skin necrosis and focal implant exposure in the lower inner quadrant.

Preoperatively, the anterior intercostal artery was identified with ultrasound in the upright position, and the flap was designed around its vascular pedicle. The flap was based on a perforator arising from the anterior intercostal artery system, which originates from the internal mammary vessels and provides reliable vascular supply to the inframammary region. The AICAP flap uses perforators from either the muscular or rectal segment of intercostal vessels. Radiological studies have revealed that the most numerous major perforators are located in the lateral third.^11,12^ In our case, 2 major perforators were identified with Doppler ultrasound, and the flap was ultimately based on the most reliable lateral perforator. The IMF was marked as the superior border, whereas the inferior border was determined with a pinch test. The medial border followed the breast axis, and the lateral extent was between the anterior and posterior axillary lines, with tapered markings to allow closure without dog ears (Figure 1).

**Figure 2.**
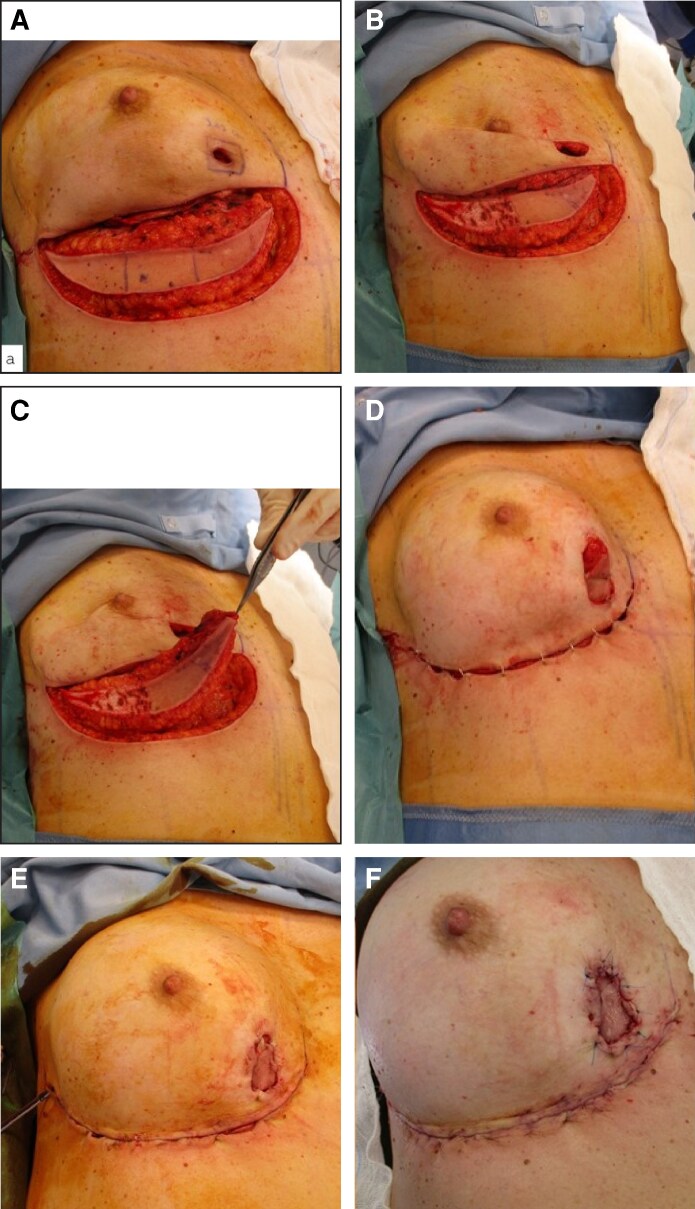
Intraoperative view of anterior intercostal artery perforator flap elevation and inset in a 54-year-old female patient. (A-C) Elevation of the fully islanded anterior intercostal artery perforator flap in the subfascial plane. (D) Flap rotation superiorly to cover the defect after implant replacement. (E, F) Immediate postoperative appearance following layered closure.

Under general anesthesia and antibiotic prophylaxis, the flap was elevated from the sixth intercostal space. After full-thickness skin incisions along the markings, it was raised in a subfascial plane from lateral to medial. A 1.5 cm skin island was preserved to cover the dehiscent area, whereas the remaining flap was de-epithelialized for insetting. Pulsatility of the perforator was manually assessed, and partial dissection was performed to allow adequate mobility and a broad arc of rotation. Key steps of flap planning, elevation, and inset are demonstrated in Figure 2.

The exposed implant was removed, and a culture was taken (later negative). The pocket was irrigated with saline and povidone–iodine, and a new implant of the same size and type was inserted. The flap was rotated superiorly to cover the defect and secured in place, preserving vascular integrity. A drain was positioned, and the wound was closed in layers.

### Postoperative Care and Outcomes

The postoperative course was uneventful. No additional antibiotics were required given the absence of infection. The drain was removed on postoperative Day 4.

**Figure 3.**
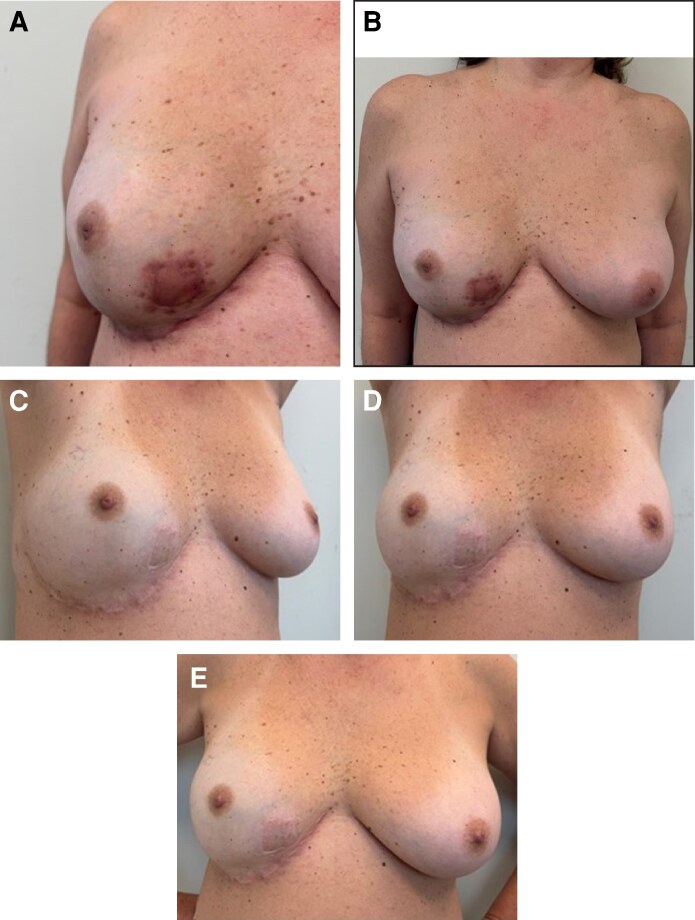
Postoperative outcome following implant salvage using an anterior intercostal artery perforator flap in a 54-year-old female patient. (A, B) Early postoperative result demonstrating flap viability. (C-E) Six-month follow-up showing stable coverage and satisfactory aesthetic outcome with mild inframammary fold blunting.

At 2 weeks, the flap was viable with no signs of infection or wound complications. By 3 months, complete healing was achieved without seroma or capsular contracture. At 6 months, the flap was fully integrated, with favorable aesthetic results and high patient satisfaction. Mild blunting of the IMF was observed but did not result in functional complaints or difficulty with undergarments (Figure 3).

## DISCUSSION

IE after mastectomy remains a major reconstructive challenge, often leading to implant loss and delayed secondary surgery.^2^

The AICAP flap was first characterized by Hamdi et al as an effective reconstructive option for sternal defects, underscoring its role in complex chest wall repair.^13^ Hallock further emphasized its versatility by describing a comparable flap for local wound management.^14^ In 2017, Carrasco-López et al reported successful use of this flap in 14 patients after breast-conserving surgery, supported by anatomical and radiological studies mapping intercostal perforators along the IMF.^15^ These early reports laid the foundation for the subsequent extension of the AICAP flap to breast reconstructive surgery, where its reliable vascularity and pliability offer significant advantages for implant salvage procedures. More recently, Mesa et al reported the use of intercostal artery perforator flaps for salvage breast reconstruction in the setting of IE, demonstrating favorable outcomes in selected patients.^16^ Similarly, Marruzzo et al described new applications of the AICAP flap in prosthetic breast reconstruction, particularly in patients undergoing radiotherapy.^17^ These reports further support the versatility of this flap in complex reconstructive scenarios.

Compared with local advancement or regional flaps, the AICAP flap offers several advantages: reliable perfusion, technical simplicity, and low donor-site morbidity.^8^ Although the AICAP flap has been previously described for breast reconstruction and implant salvage, reports specifically addressing delayed IE following prepectoral reconstruction remain limited, and standardized management algorithms are lacking.^16,17^ In this setting, reconstructive options are often restricted, and implant removal is frequently recommended. The present case highlights the role of the AICAP flap as a minimally invasive alternative that may allow implant preservation in carefully selected patients.

This case demonstrates that even in a compromised setting, with a history of smoking and endocrine therapy, the AICAP flap can achieve successful reconstruction salvage. Its use preserves the reconstructive investment while avoiding more complex or morbid secondary surgeries. A mild blunting of the IMF was observed postoperatively, which represents a known potential trade-off when recruiting tissue from the IMF region. In our patient, this did not translate into functional discomfort or dissatisfaction, but careful preoperative counseling and meticulous flap design are essential to minimize this risk. Although a degree of IMF blunting may occur after implant salvage with a local perforator flap, the primary reconstructive goal in our case remains stable implant coverage. We reconstructed the IMF intraoperatively using fixation of the abdominal tissue to the chest wall in order to recreate the fold. Additional aesthetic refinements may be required, and the IMF may be reliably redefined with a secondary dermofascial fixation suture or secondary lipofilling.

The use of PU-coated implants was also advantageous in our case.

Several studies have demonstrated that PU-coated implants allow for effective containment of the defect and reduction of dead space, thereby lowering the incidence of seroma and, consequently, the risk of infection.^18^ The PU coating adheres rapidly to surrounding tissues (Velcro effect), obliterating potential spaces. In a comparative prepectoral cohort, seromas and infections occurred only in the ADM group, with no cases reported in the PU implant group. The authors attributed the adhesive properties of PU to decreased fluid accumulation and, as a result, to a lower risk of infection and reconstructive failure.^10^

In our case, the use of a PU-coated implant allowed immediate replacement while maintaining breast volume and potentially reducing the risk of fluid-related complications.

### Limitations

This report has inherent limitations because it describes a single case with a relatively short follow-up. Furthermore, patient selection plays a crucial role in determining the success of implant salvage, and this approach may not be suitable in cases of extensive infection, radiotherapy-related tissue damage, or systemic compromise. Larger series are needed to better define indications and long-term outcomes.

## CONCLUSIONS

The AICAP flap is a reliable and effective option for implant salvage in breast reconstruction, particularly in cases of localized complications without systemic infection. This technique helps avoid implant removal and more complex secondary reconstruction, whereas the use of PU-coated implants may further enhance outcomes. Larger studies are warranted to confirm long-term efficacy and refine indications.
